# Standardizing Chemotherapy Regimen Nomenclature: A Proposal and Evaluation of the HemOnc and National Cancer Institute Thesaurus Regimen Content

**DOI:** 10.1200/CCI.19.00122

**Published:** 2020-01-28

**Authors:** Samuel M. Rubinstein, Peter C. Yang, Andrew J. Cowan, Jeremy L. Warner

**Affiliations:** ^1^ Division of Hematology and Oncology, Department of Medicine, Vanderbilt University Medical Center, Nashville, TN; ^2^ Department of Biomedical Informatics, Vanderbilt University Medical Center, Nashville, TN; ^3^ Division of Hematology/Oncology, Department of Medicine, Massachusetts General Hospital, Boston, MA; ^4^ Division of Medical Oncology, Department of Medicine, University of Washington, Seattle, WA

## Abstract

**PURPOSE:**

Due to decades of nonstandardized approaches to the naming of chemotherapy regimens, representation in electronic health records and secondary systems is highly variable. This hampers efforts to understand patterns of chemotherapy usage at the population level. In this article, we describe a proposal for rules to standardize the nomenclature of chemotherapy regimens and illustrate applications of these rules.

**METHODS:**

Through our experience with building HemOnc.org, which has been under construction since 2011, we formulated a set of guidelines and recommendations for the standard representation of chemotherapy regimen names. We then performed a mapping between the HemOnc and National Cancer Institute Thesaurus vocabulary’s regimens and evaluated conformance with the naming conventions. Finally, we assembled a database of acronyms and names for multiple myeloma regimens to illustrate the scope of the problem.

**RESULTS:**

For the first use case, 242 of 527 (45.1%) of the regimen names differed. The schema was able to allocate a preferred source for 217 (89.4%) of these regimens. For the second use case, we expanded 130 multiple myeloma regimens to 1,138 unique regimen names and demonstrate ways in which the schema can collapse these into disambiguated, but abbreviated, regimen names.

**CONCLUSION:**

To our knowledge, this is the first proposal to normalize chemotherapy regimen nomenclature. If our recommendations are adopted, we expect that the uniformity of treatment exposure representation in hematology/oncology will increase, which will enable large-scale efforts such as ASCO’s CancerLinQ to achieve better standardization.

## INTRODUCTION

The introduction of combination chemotherapy in the 1960s and 1970s has led to ever-increasing complexity of cancer treatment regimens. On the basis of the success of certain combinations in curing certain cancers, multidrug combination regimens, often with complex sequencing and dosing parameters, have become common. As a result, an informal jargon based primarily on acronyms arose to facilitate communication between oncologists and with patients. This jargon is idiosyncratic and can include a mix of generic, chemical, and brand names of antineoplastics. For example, the lymphoma regimen CHOP, originally named the uninformative “Combination 2,” contains two generic abbreviations (C for cyclophosphamide and P for prednisone/prednisolone), one brand-name abbreviation (O for Oncovin [vincristine]), and one chemical name abbreviation (H for hydroxydaunorubicin/hydroxyldaunomycin).^1-3^ The regimen PAD contains one generic abbreviation (D for dexamethasone), one brand-name abbreviation (A for Adriamycin [doxorubicin]), and one code name abbreviation (P for PS-341 [bortezomib]).^4^ As the majority of bortezomib-containing regimens abbreviate it with a “V” (its brand name is Velcade), PAD is sometimes called VAD in practice. This creates confusion with an older regimen that was a standard of care for many years, VAD,^5^ where V stands for vincristine (as opposed to Velcade [bortezomib]).

As drug development accelerates, and an increasing armamentarium of antineoplastics is incorporated into treatment regimens, examples such as this will become increasingly common. The oncology community needs a lingua franca for documenting the consideration, planning, administration, and history of chemotherapy regimens. There are many potential downsides to the current state of nonstandardized representation. Most importantly, clinical care can be affected. For example, a tertiary referral center might misinterpret the “VAD” in a patient’s treatment history as having contained bortezomib and might consider the patient exposed and resistant when they are actually bortezomib naïve. In addition to facilitating better communication between clinicians, a standardized regimen-naming convention would facilitate secondary use of treatment exposure data by external registrars and learning health systems such as CancerLinQ and for studies using electronic health record (EHR) data. At least in part because of difficulties with regimen naming, the cancer registry community has historically only recorded individual drug exposures, when they are recorded at all. This can lead to ambiguity and insufficient detail to understand patterns of treatment exposure. For example, R-EPOCH^6^ and dose-adjusted R-EPOCH^7^ contain the same drugs but are distinct in terms of doses, indications, administration times, and other details. The predictable result is chaos when approaching the problem of aggregating real-world data at a population scale, as is envisioned by the US Food and Drug Administration (FDA)^8^ as well as efforts such as ASCO’s CancerLinQ,^9^ Flatiron, and American Association for Cancer Research Genomics Evidence Neoplasia Information Exchange (GENIE).^10^

**CONTEXT**

**Key Objective**
The aim of this work was to develop the first standardized nomenclature, to our knowledge, for representation of chemotherapy regimens, which have been long recognized to have ambiguous and nonstandardized naming conventions.
**Knowledge Generated**
Rules for the representation of chemotherapy regimens as standardized concepts were developed. These rules were applied to map regimens represented by HemOnc.org and the National Cancer Institute Thesaurus and to adjudicate preferred regimen representations, as well as to demonstrate the degree of ambiguity in regimen representation for multiple myeloma regimens.
**Relevance**
If adopted, this nomenclature has the potential to improve uniformity in representation of treatment concepts, facilitating large-scale data analysis for learning health systems and electronic health record–based investigation.

Informed primarily by our experience building HemOnc.org,^11^ the largest freely available resource of chemotherapy drug and regimen information, we have introduced the first standardized vocabulary for chemotherapy regimens in the Observational Medical Outcomes Partnership common data model, allowing for transformation of data derived from distinct observational sources into a uniform format.^12^ Here, we take this further, proposing a standard nomenclature for chemotherapy regimens. What follows is inspired by the model of the desiderata proposal of Cimino,^13^ which establishes criteria for standardizing medical vocabularies including unique identification of concepts, concept permanence, and orientation. It is meant to spark a discussion within the communities of clinicians (clinical document creators), guideline creators, and clinical data consumers (secondary users of EHR data). We propose 13 rules for naming chemotherapy regimens on the basis of our experience curating HemOnc.org and our day-to-day experience as clinicians. Subsequently, we evaluate the scope of the current challenge in the area of multiple myeloma and perform a mapping and comparison of the HemOnc^12^ and National Cancer Institute Thesaurus (NCIT)^14^ content.

## NOMENCLATURE PROPOSAL

We propose a schema for standardized nomenclature of categories of chemotherapy regimens, organized in increasing order of regimen complexity. Our recommendations use the conformance verbs SHALL, SHOULD, and MAY as defined in Network Working Group Request for Comment 2119.^15^ Relevant examples are given in Table 1.

**TABLE 1.**
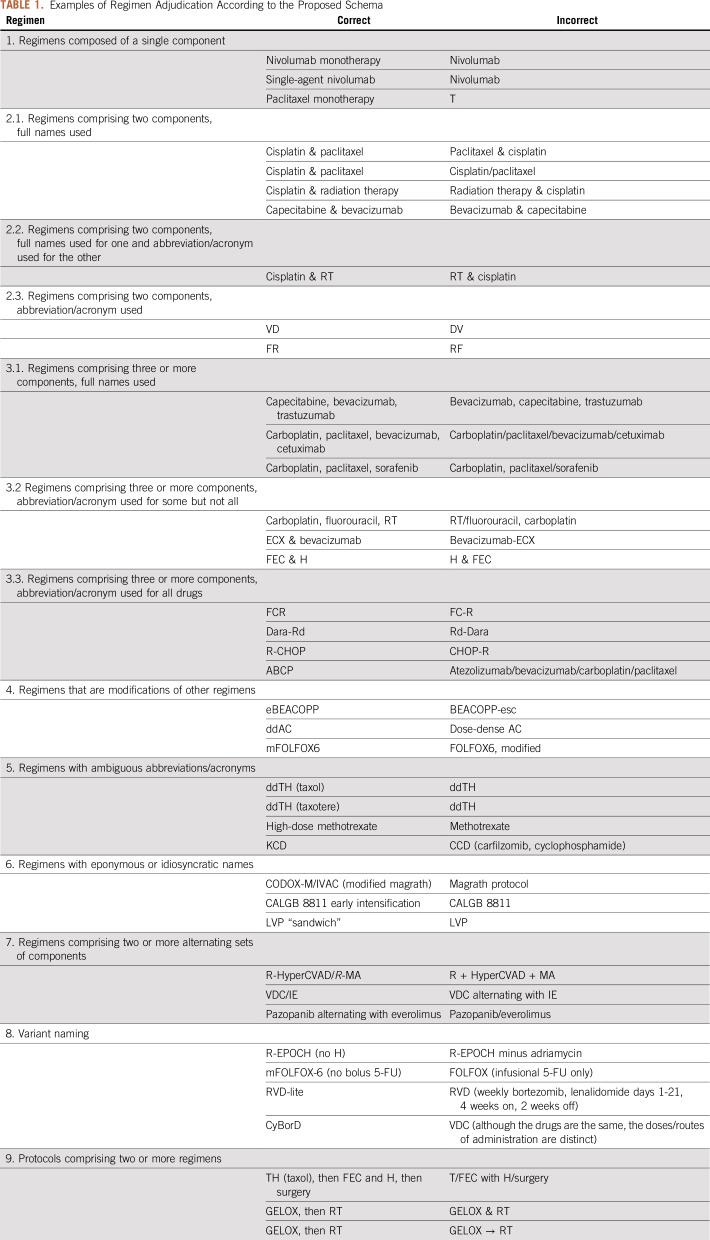
Examples of Regimen Adjudication According to the Proposed Schema

### 1. Regimens Composed of a Single Component

These shall be expressed by the generic drug name followed by the suffix “monotherapy.” Alternatively, they can be expressed by the prefix “single-agent” followed by the component name. The suffix “monotherapy” is preferred, unless referring to a category (eg, “single-agent chemotherapy”). Using acronyms to represent a single component should be avoided, as single letters will be frequently confused with other EHR artifacts.

### 2.1. Regimens Comprising Two Components, Full Names Used

When the two components are of the same drug category, these shall be expressed in alphabetical order of the generic name, separated by “and” or “&.” When one of the components is a cytotoxic and the other is a monoclonal antibody, a steroid, or radiation, the cytotoxic shall be expressed first. For each component, only the first word is capitalized.

### 2.2. Regimens Comprising Two Components, Full Name Used for One and Abbreviation/Acronym for the Other

This is generally discouraged except for one case: the abbreviated component is radiation therapy (RT). In this case, the rule is the same as above: “Cisplatin & RT” is correct; “RT & Cisplatin” is not.

### 2.3. Regimens Comprising Two Components, Abbreviation/Acronym Used

These are particularly prone to ambiguity. For example, the small-cell lung cancer (SCLC) regimen comprising cisplatin and etoposide has been variably called EC (etoposide & cisplatin), EP (etoposide & Platinol [cisplatin]), and PE (Platinol [cisplatin] & etoposide). In these cases, it is probably best to expand the acronym and follow the rules outlined in 2.1. Otherwise, there are four guidelines to consider:

1. Avoid acronyms that are easily confused with other medical acronyms (eg, PE is more commonly an abbreviation for pulmonary embolism and EP is an abbreviation for electrophysiology).
2. Avoid acronyms that use a brand name (eg, EP or PE).
3. Avoid acronyms that are much more commonly used for other regimens (eg, EC is a common acronym for the breast cancer regimen of epirubicin and cyclophosphamide).
4. Use the acronym that is most often used in the published literature.

These guidelines can be in direct conflict. For example, a Google Scholar search for “EC SCLC chemotherapy,” “EP SCLC chemotherapy,” and “PE SCLC chemotherapy” yields 8,580, 12,200, and 11,100 results, respectively. Comparatively, a Google Scholar search for “EC breast cancer chemotherapy” yields 357,000 results; as such, acronym EC should be reserved for the breast cancer regimen, and, given the violation of rules 1, 2, and the relative inconclusiveness of rule 4, the drug names cisplatin and etoposide should be used instead of the acronyms PE or EP.

### 3.1. Regimens Comprising Three or More Components, Full Names Used

Components shall be separated by a comma, without the use of “and” (eg, “A, B, C, D” not “A, B, C, and D”). Components shall be listed alphabetically within subcategory, as follows: cytotoxics first, then monoclonal antibodies, then steroids.

### 3.2. Regimens Comprising Three or More Components, Abbreviation/Acronym Used for Some but Not All

There are two cases where this is acceptable:

1. As before, the component of radiation therapy can and should be abbreviated as RT.
2. If two or more components compose a well-known “backbone” that is conventionally referred to by acronym.

For regimens containing radiation therapy, the acronym should be listed last (eg, Carboplatin, Fluorouracil, RT). For regimens containing “backbones” represented by acronyms (eg, ECX & bevacizumab), the acronym should be listed first; the additional component should be listed last. As in section 3.1, components or acronyms shall be separated by a comma, without the use of “and” if there are three or more components, and by “and” or “&” if there are two components or acronyms.

### 3.3. Regimens Comprising Three or More Components, Abbreviation/Acronym Used for All Drugs

In many cases, a philosophical disagreement exists over whether abbreviating a regimen is necessary or advisable for a particular regimen. Exchanging more-precise for less-precise terminology always introduces potential ambiguity. There is no a priori way to adjudicate this issue, as the specifics of individual regimens are important. However, shortening the names of regimens potentially reduces documentation burden for clinicians and registrars, especially when regimens contain more than three drugs or involve alternating combinations of chemotherapy. Regimens should therefore be abbreviated in some form when it is possible to do so in a way that clearly conveys the treatment protocol being used for a particular patient.

In contrast to the situation described in rule 2.3, ambiguity is less common in this setting, but it still exists. R-CHOP is a concrete example; 3,952 patients at Vanderbilt University Medical Center have the acronyms “R-CHOP,” “RCHOP,” “CHOP-R,” or “CHOPR”—representing the chemotherapy regimen of rituximab, cyclophosphamide, doxorubicin, vincristine, and prednisone—present in their EHR. In this case a Google Scholar search is informative; there are 24,100 results for “R-CHOP chemotherapy,” 6,060 for “CHOP-R chemotherapy,” 2,230 for “RCHOP chemotherapy,” and 64 for “CHOPR chemotherapy.” R-CHOP is preferred.

Additional ambiguity arises when a biologic agent is added to a chemotherapy backbone and is itself abbreviated. In many cases, the abbreviated biologic agent is affixed on the front end (R-CHOP, *R*-GemOx); when this is done, it is typically separated from the chemotherapy backbone by hyphenation. In other cases, the approved biologic agent is affixed on the back end (BR, FCR), in which case it is often added to the chemotherapy backbone with no separation. Empirically, we find that this convention generally holds, and we propose that it be followed for novel analogous combinations.

The authors of a paper often specify an abbreviation for a particular regimen; in these cases, as long as this does not conflict with an abbreviation for an existing regimen, it is advisable to use this abbreviation, even if it does not hold to the conventions specified herein. This facilitates linkage of a regimen described in clinical documentation or a tumor registry with a corresponding reference in the literature.

### 4. Regimens That Are Modifications of Other Regimens

Several commonly used regimens are in fact modifications of older regimens that have overtaken their predecessors in clinical use, usually because of superiority in a randomized trial. We propose the following shorthand nomenclature to indicate the type of modification: e for escalated, m for modified, dd for dose-dense, di for dose-intense, and da for dose-adjusted. If a particular intensity of a drug is used, “high-dose” or “low-dose” are acceptable modifiers.

### 5. Regimens With Ambiguous Abbreviations/Acronyms

If one of the letters of an abbreviation or acronym is likely to be confused with a similar component of another regimen, the name of the abbreviated component should be provided in parentheses immediately after the regimen name.

In some cases, multiple components of a regimen may be abbreviated identically, in which case clarifying the ambiguous agent parenthetically requires doing so multiple times. For some of these, one of the agents has an alternate or brand name that can be abbreviated distinctly. Although generic names are preferable in the vast majority of cases, this represents an exception in which a brand name is preferred (Table 2).

**TABLE 2.**
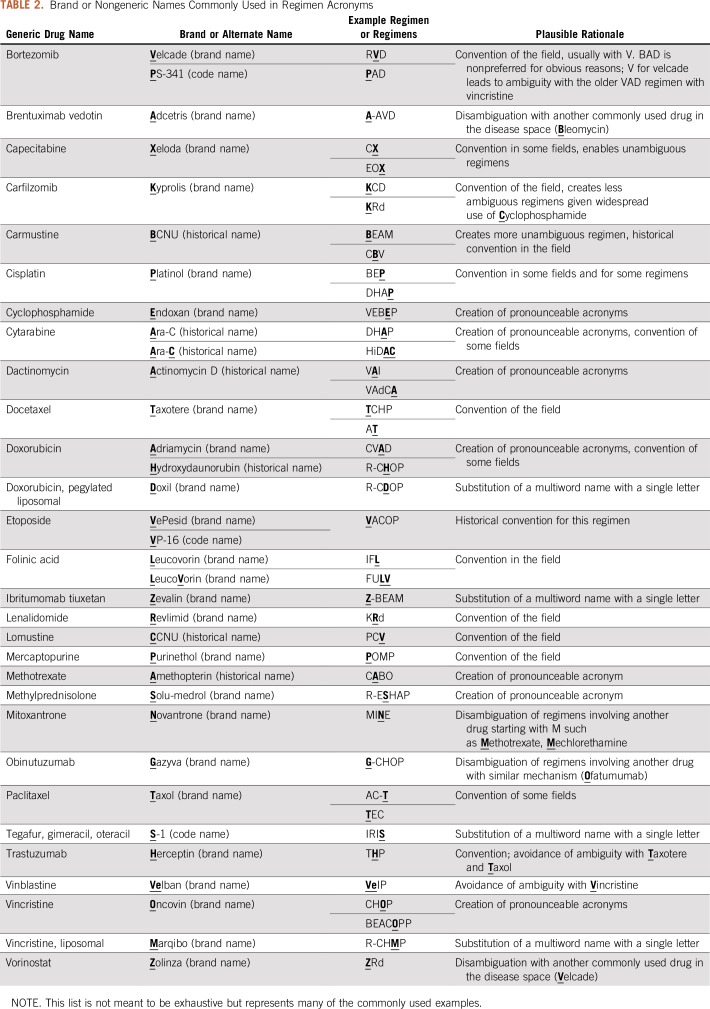
Brand or Nongeneric Names Commonly Used in Regimen Acronyms

Wherever possible, a regimen should not be abbreviated in such a way that makes it identical to an English word or nonchemotherapy medical abbreviation.

### 6. Regimens With Eponymous or Idiosyncratic Names

Although generally to be avoided, some regimens and protocols are very well established by the name of an individual or a group study. In these cases, if there is a readily understood noneponymous regimen name it should be used, with the eponym parenthetically immediately after the regimen name. If no such nomenclature readily exists, it should be indicated as a regimen, either by inclusion of the word “regimen” or with alternate modifiers clarifying the preceding acronym. This is especially important when considering protocols involving many distinct regimens administered over months to years (see section 9 for further discussion).

### 7. Regimens Comprising Two or More Alternating Sets of Components

These regimens consist of subsets of regimens that cannot be inextricably unlinked because of the alternation schedule (eg, A is followed by B, then A, then B, then A, then B). In these cases, when the subsets are acronyms, individual subsets should be separated by a “/”. The “/” indicator shall be reserved for this purpose only; using it elsewhere creates ambiguity as to whether the drugs separated by “/” are given sequentially or together. If the subsets are full names, the separator should be “alternating with.”

### 8. Variant Naming

Many widely used regimens are variants of other regimens, involving the same drugs on an alternate dose level or schedule that does not easily fit into the modification paradigm as explained above. Sometimes these variants exist to avoid toxicity in the context of antecedent organ dysfunction or to treat patients who were too frail to be studied in initial trials. If such variants are indicated by widely accepted modifiers, these should be used, as they can avoid significant complexity in regimen naming while conveying the same meaning. If not, the modification to the existing regimen should be indicated as succinctly as possible, with the modified drug indicated in the same fashion as in the initial regimen.

### 9. Protocols Comprising Two or More Regimens

These are often complex treatments that consist of two or more sequential procedures or regimens assembled into a total protocol. Each temporally distinct regimen or procedure should be separated by “, then.” There are some exceptions to this; for example, some widely used sequential breast cancer regimens involve a sequence of an anthracycline/cyclophosphamide doublet and a taxane, and the temporal separation between regimens is represented by a dash (eg, AC-T). Unless such representations are nearly ubiquitous, they should be avoided.

## EVALUATION

### Use Case 1: NCIT to HemOnc Regimen Mapping

We manually mapped the preferred distinct regimen names in the HemOnc vocabulary to the corresponding preferred concepts in the NCIT. The purpose of this was two-fold: 1) providing additional external mappings in HemOnc (eg, procedures are mapped to Systematized Nomenclature of Medicine-Clinical Terms [SNOMED-CT]; drugs to RxNorm; and conditions to NCIT and SNOMED-CT), and 2) determining the degree to which the preferred names in the vocabularies align with these proposed rules.

**FIG 1.**
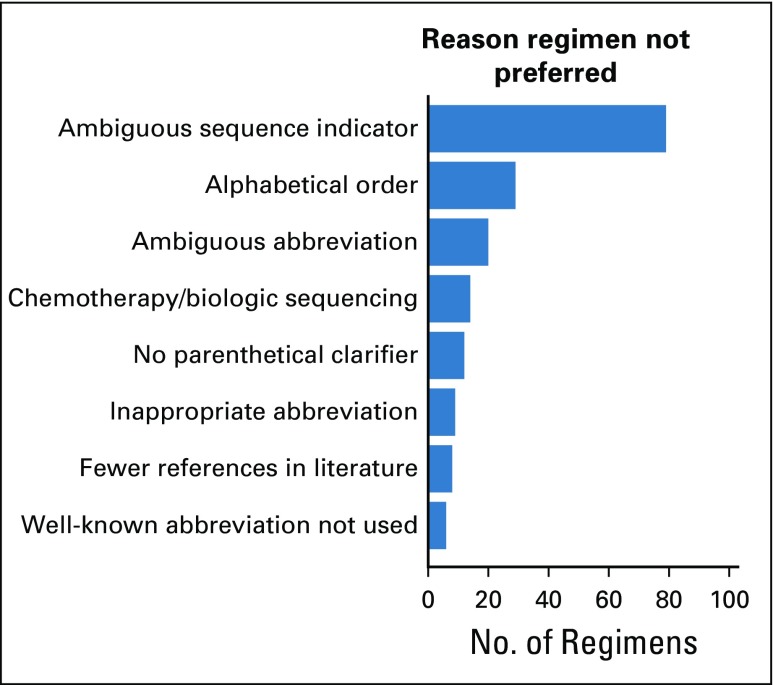
Discrepancy analysis of HemOnc and National Cancer Institute Thesaurus preferred names. The reason for names not being preferred follows the examples shown in Table 1.

There were 527 successfully mapped regimens; of these 285 (54.1%) are described identically on NCIT, when accounting for semantic differences in punctuation and labeling concepts as “regimens” (Data Supplement). This leaves 242 (45.9%) in which the same concept is represented divergently by the two databases. Of these, the schema above was able to identify a clearly preferable regimen for 217 (90.0%); the HemOnc nomenclature was preferred in 194 cases (89.4%), and the NCIT nomenclature was preferred in 23 cases (10.6%). Rationales for allocating a preferable regimen are summarized in Figure 1.

**FIG 2.**
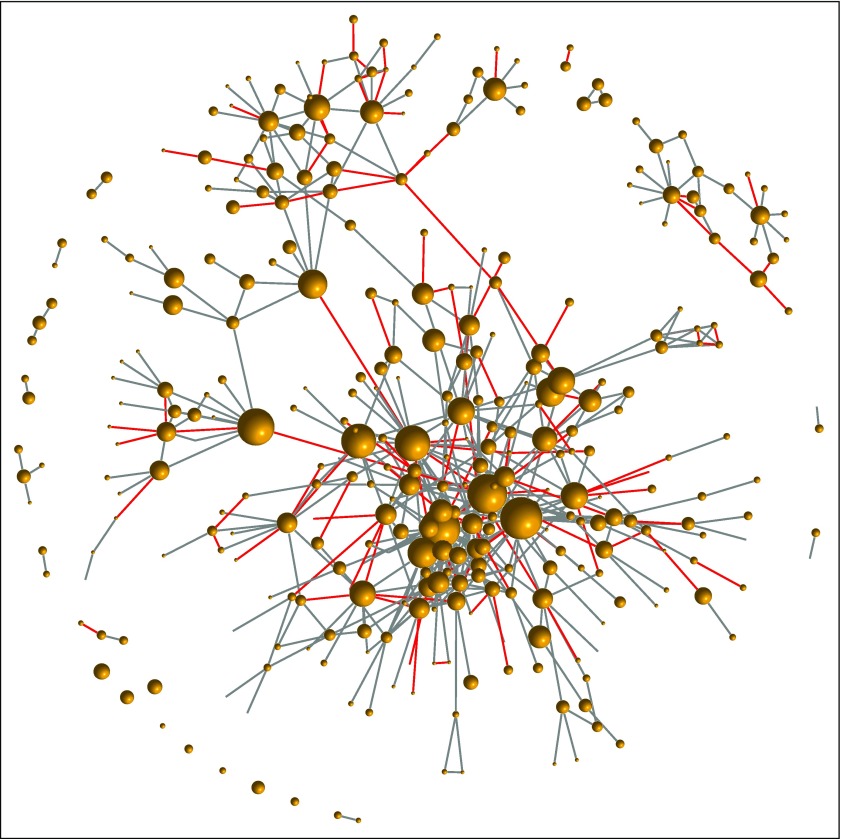
Example of layering a HemOnc concept onto the mapped regimens. Each vertex in the graph represents a regimen, and edges represent at least one instance of the “Has been compared to” relationship in HemOnc. Node size is proportionate to the number of studies involving the regimen as a comparator (eg, the largest node is docetaxel monotherapy, with N = 118 studies). Edges colored red represent randomized clinical trial comparisons that were the basis of a US Food and Drug Association drug approval or new indication. The HemOnc vocabulary is available to academic and noncommercial users through the CC BY-NC-SA 4.0 license.

For 25 discrepancies, the proposed rules could not allocate a preferred name. The two sources generally chose ways to abbreviate regimens that were equally unambiguous and potentially useful, are distinct, and have similar representation in the literature. It is challenging to adjudicate these cases until one particular nomenclature is adopted more widely. Beyond the nomenclature evaluation, this mapping offers utility to the community. For example, native NCIT users can layer HemOnc relationships into their applications (Fig 2).

### Use Case 2: Multiple Myeloma Regimen Nomenclature

The magnitude of this problem truly comes into focus when examining the landscape of multiple myeloma regimens. There are 17 FDA-approved drugs for the treatment of this malignancy, 7 of which were approved in 2012 or later; more than 100 regimens have been evaluated in multiple myeloma in phase II or III studies. Several recent randomized controlled trials have demonstrated superior progression-free survival for three-drug combinations compared with two drug combinations in a variety of treatment settings.^16-18^ Four-drug combinations are currently under active investigation.^19^ Because of these developments, increasingly complex regimens combining chemotherapeutic agents and monoclonal antibodies are becoming the standard of care. An additional complication arises because lenalidomide plus high-dose dexamethasone has been compared with lenalidomide plus low-dose dexamethasone.^20^ These are different regimens and are often represented distinctly (“RD” for high-dose dexamethasone, “Rd” for low-dose dexamethasone). One can imagine that representation of these regimens is not homogeneous, both in the literature and in clinical documentation. Some examples of variants of commonly used regimens are listed in Table 3.

**TABLE 3.**
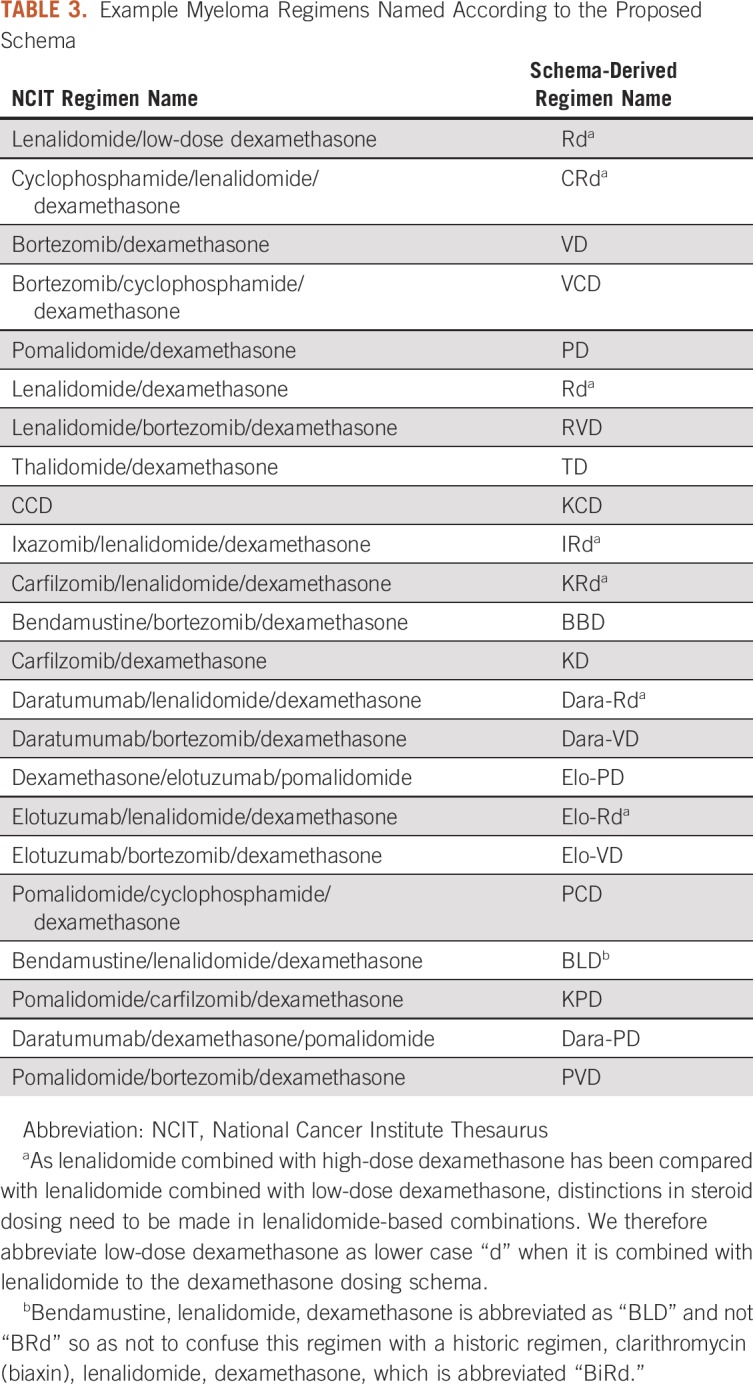
Example Myeloma Regimens Named According to the Proposed Schema

We reviewed the 130 multiple myeloma regimens contained within HemOnc. On the basis of the representation of these regimens in the literature and our empirical experience in clinical practice, we have recorded 1,138 acronyms representing these regimens (Data Supplement). This is not intended to be comprehensive but nevertheless illustrates the scope of the problem. Individual regimens have a median of six distinct representations, with a maximum of 31 for one four-drug combination (bortezomib, cyclophosphamide, lenalidomide, and dexamethasone). This heterogeneity in representation creates challenges in determining prior treatment exposure, and therefore eligibility for subsequent therapy at relapse, which is inevitable for this incurable malignancy.

Using the rules-based approach for uniform nomenclature, we collapsed these myeloma regimens into representations fitting our schema (Table 4).^21^ Although alternate representations for these concepts exist, this approach does so in a way that allows significant abbreviation and minimal ambiguity as to the regimen being used. To our knowledge, this is the first proposal for a schema to normalize chemotherapy regimen nomenclature. We have demonstrated with two use cases that this schema is useful to disambiguate regimen representation. Our schema can be used to adjudicate differences in regimen representation and can collapse an increasingly complex landscape of regimen representation into a short, digestible list of concepts.

**TABLE 4.**
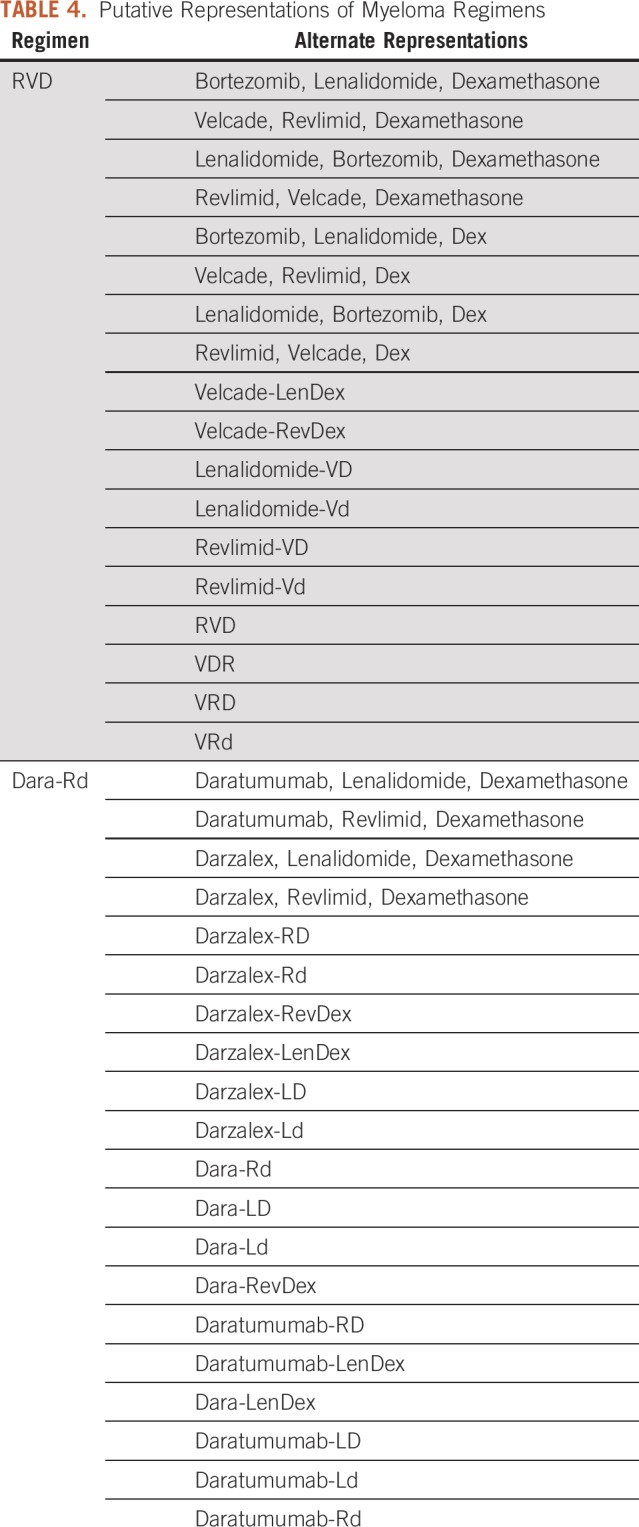
Putative Representations of Myeloma Regimens

We acknowledge that different potential methodologies exist to standardize chemotherapy regimen representation. We do not claim or aim to demonstrate that our proposal is definitively superior to possible alternatives. Because no standardization schema yet exists, were the standard presented in this paper adopted, the uniformity of treatment representation in the literature and in clinical documentation could increase. As regimens grow more complex and involved over time, it is becoming increasingly important to develop a priori best practices for naming new regimens. Adopting a standard nomenclature, whether the one proposed here or an alternative, would facilitate this. We hope that this effort stimulates discussion surrounding standardization of chemotherapy nomenclature. This would facilitate a variety of efforts, including knowledge aggregation, real-world data analysis, and developing an oncology learning health system.

## References

[1] Gottlieb JA Gutterman JU McCredie KB et al Chemotherapy of malignant lymphoma with adriamycin Cancer Res 33 3024 3028 1973 4748450

[2] Elias L Portlock CS Rosenberg SA Combination chemotherapy of diffuse histiocytic lymphoma with cyclophosphamide, adriamycin, vincristine and prednisone (CHOP) Cancer 42 1705 1710 1978 361209 10.1002/1097-0142(197810)42:4<1705::aid-cncr2820420408>3.0.co;2-p

[3] McKelvey EM Gottlieb JA Wilson HE et al Hydroxyldaunomycin (Adriamycin) combination chemotherapy in malignant lymphoma Cancer 38 1484 1493 1976 791473 10.1002/1097-0142(197610)38:4<1484::aid-cncr2820380407>3.0.co;2-i

[4] Oakervee HE Popat R Curry N et al PAD combination therapy (PS-341/bortezomib, doxorubicin and dexamethasone) for previously untreated patients with multiple myeloma Br J Haematol 129 755 762 2005 15953001 10.1111/j.1365-2141.2005.05519.x

[5] Barlogie B Smith L Alexanian R Effective treatment of advanced multiple myeloma refractory to alkylating agents N Engl J Med 310 1353 1356 1984 6546971 10.1056/NEJM198405243102104

[6] Jermann M Jost LM Taverna Ch et al Rituximab-EPOCH, an effective salvage therapy for relapsed, refractory or transformed B-cell lymphomas: Results of a phase II study Ann Oncol 15 511 516 2004 14998858 10.1093/annonc/mdh093

[7] Wilson WH Dunleavy K Pittaluga S et al Phase II study of dose-adjusted EPOCH and rituximab in untreated diffuse large B-cell lymphoma with analysis of germinal center and post-germinal center biomarkers J Clin Oncol 26 2717 2724 2008 18378569 10.1200/JCO.2007.13.1391 PMC2409217

[8] Khozin S, Blumenthal GM, Pazdur R: Real-world data for clinical evidence generation in oncology. J Natl Cancer Inst 109:djx187, 2017 10.1093/jnci/djx187 29059439

[9] Rubinstein SM, Warner JL: CancerLinQ: Origins, implementation, and future directions. JCO Clin Cancer Inform 10.1200/CCI.17.00060 10.1200/CCI.17.00060 30652539

[10] AACR Project GENIE Consortium AACR project GENIE: Powering precision medicine through an international consortium Cancer Discov 7 818 831 2017 28572459 10.1158/2159-8290.CD-17-0151 PMC5611790

[11] Warner JL Cowan AJ Hall AC et al HemOnc.org: A collaborative online knowledge platform for oncology professionals J Oncol Pract 11 e336 e350 2015 25736385 10.1200/JOP.2014.001511 PMC5706141

[12] Warner JL Dymshyts D Reich CG et al HemOnc: A new standard vocabulary for chemotherapy regimen representation in the OMOP common data model J Biomed Inform 96 103239 2019 31238109 10.1016/j.jbi.2019.103239 PMC6697579

[13] Cimino JJ Desiderata for controlled medical vocabularies in the twenty-first century Methods Inf Med 37 394 403 1998 9865037 PMC3415631

[14] Sioutos N de Coronado S Haber MW et al NCI Thesaurus: A semantic model integrating cancer-related clinical and molecular information J Biomed Inform 40 30 43 2007 16697710 10.1016/j.jbi.2006.02.013

[15] https://tools.ietf.org/html/rfc2119.

[16] Dimopoulos MA Moreau P Palumbo A et al Carfilzomib and dexamethasone versus bortezomib and dexamethasone for patients with relapsed or refractory multiple myeloma (ENDEAVOR): A randomised, phase 3, open-label, multicentre study Lancet Oncol 17 27 38 2016 26671818 10.1016/S1470-2045(15)00464-7

[17] Durie BGM Hoering A Abidi MH et al Bortezomib with lenalidomide and dexamethasone versus lenalidomide and dexamethasone alone in patients with newly diagnosed myeloma without intent for immediate autologous stem-cell transplant (SWOG S0777): A randomised, open-label, phase 3 trial Lancet 389 519 527 2017 28017406 10.1016/S0140-6736(16)31594-X PMC5546834

[18] Facon T Kumar S Plesner T et al Daratumumab plus lenalidomide and dexamethasone for untreated myeloma N Engl J Med 380 2104 2115 2019 31141632 10.1056/NEJMoa1817249 PMC10045721

[19] Voorhees PM Rodriguez C Reeves B et al Efficacy and updated safety analysis of a safety run-in cohort from griffin, a phase 2 randomized study of daratumumab (Dara), bortezomib (V), lenalidomide (R), and dexamethasone (D; Dara‐Vrd) vs. Vrd in patients (Pts) with newly diagnosed (ND) multiple myeloma (MM) eligible for high‐dose therapy (HDT) and autologous stem cell transplantation (ASCT) Blood 132 2018 suppl 1; abstr 151

[20] Rajkumar SV Jacobus S Callander NS et al Lenalidomide plus high-dose dexamethasone versus lenalidomide plus low-dose dexamethasone as initial therapy for newly diagnosed multiple myeloma: An open-label randomised controlled trial Lancet Oncol 11 29 37 2010 19853510 10.1016/S1470-2045(09)70284-0 PMC3042271

[21] Rajkumar SV: Multiple myeloma: 2018 update on diagnosis, risk‐stratification, and management. Am J Hematol 93:1091-1110, 2018

